# Homœopathy and Its Relation to the Germ Theory

**Published:** 1885-09

**Authors:** A. Lippe


### Homœopathy and its Relation to the Germ Theory. By Robert N. Tooker, M. D. Being the President's Address delivered before the Illinois Homœopathic Medical Association, at Peoria, Ill., May 29th, 1885. Gross & Delbridge, Chicago, 1885.

The most lucid exposition of this subject, *Homœopathy and its Relation to the Germ Theory*, is to be found in this most excellent address. Hahnemann was the first healer who opened the way for a "*certainty in medicine*." He succeeded in demonstrating practically the application of the law of the similars for the cure of the sick. The Allopathic School of Medicine has offered various and ever-changing theories as to the nature and causes of diseases. Each new theory found its adherents and defenders because the previous theories proved themselves to be erroneous when applied practically. The latest theory is the Germ Theory. And how does it advance the therapeutics of the old school? It has already failed when practically applied. The cholera rages in Spain and is breaking out in France. People were inoculated with Koch's cholera microbes. They were attacked by the cholera like inoculated persons and died as they did. It is strange indeed that the superior successes of the homœopathic treatment of the cholera have hardly been noticed and that this remarkably successful treatment should be ignored and a new theory be adopted which does not help in the cure of the afflicted.

As homœopathists, we cannot yet expect to draw any deductions leading to practical results from the researches of scientific men who, like Pasteur, Tyndall, and others, have tried to find the causes of human suffering. We have cured the cholera before, we will cure it again, with the similar remedy properly administered.

The isopathists have thought differently, and carried away, especially by Pasteur's experiments, have come to the illogical conclusion that the products of a disease, IF HIGHLY POTENTIZED, will cure the disease itself. Homœopathy applies the medicine which possesses similar sick-making powers to the diseased condition which manifests itself through various objective and subjective symptoms. The cholera microbes were inoculated and failed to preserve the individual from taking the disease, and if under whatever plausible or necessarily false logic the isopathists administer highly potentized microbes such therapeutics would be unsuccessful because unhomœopathic, just as the inoculation was unsuccessful because it is unhomœopathic. As homœopathists, we know that if we are true to our principles success must follow, therefore it would be absurd to set aside these our principles and methods and be led astray by novel theories not in harmony with the teachings of our school. A. LIPPE.

